# Predicting recurrence of non-muscle invasive bladder urothelial carcinoma: predictive value of the optimal cut-off value of Ki67

**DOI:** 10.3389/fonc.2024.1522009

**Published:** 2025-01-16

**Authors:** Rende Peng, Yaoyu Zhang, Mingzhu Jia, Xinping Yi, Xiaoyao Yi, Shadan Li, Jiangchuan Pi, Wenjun Meng

**Affiliations:** ^1^ Department of Urology, Chengdu Second People’s Hospital, Chengdu, China; ^2^ Department of Urology, The General Hospital of Western Theater Command, Chengdu, China; ^3^ Department of Gynecology and Obstetrics, West China Second University Hospital, Sichuan University, Chengdu, China; ^4^ Department of Urology, Yongchuan Hospital of Chongqing Medical University, Chongqing, China; ^5^ Department of Neurosurgery, The Second Affiliated Hospital of Chongqing Medical University, Chongqing, China; ^6^ Department of Biotherapy, Cancer Center, West China Hospital, Sichuan University, Chengdu, China

**Keywords:** immunohistochemical markers, ki67, non-muscular invasive bladder urothelial carcinoma, recurrence, prognosis, optimal cut-off value

## Abstract

**Objective:**

To investigate the optimal cut-off value of immunohistochemical marker Ki67 as a prognostic factor to predict the recurrence of non-muscle invasive bladder urothelial carcinoma (NMIBUC).

**Methods:**

A total of 331 patients diagnosed with NMIBUC who underwent surgery in the Yongchuan Hospital and the Second Affiliated Hospital of Chongqing Medical University from January 2012 to January 2020 were finally included in this study. The optimal cut-off value of Ki67 for predicting recurrence of NMIBUC was calculated by ROC curve and Youden index. According to the cut-off value, the patients were divided into high ratio group and low ratio group, and the clinicopathological data of the two groups were compared. Univariate and multivariate regression analysis were used to analyze the relationship between the expression of Ki67 and postoperative recurrence of NMIBUC. The Kaplan-Meier curve was used for survival analysis.

**Results:**

18% is the optimal cut-off value of Ki67 for predicting postoperative recurrence of NMIBUC. High Ki67 expression (Ki67>18%) was significantly correlated with tumor stage (P=0.001), tumor grade (P=0.014), immediate postoperative instillation (P=0.001), the expression of P53 (P=0.019) and CK20 (P=0.001). Ki67 expression greater than 18% was an independent risk factor for high recurrence rate of NMIBUC (P=0.001). Moreover, the 1-year and 3-year recurrence-free survival (RFS) of the high Ki67 group were 56.6% (95%CI 51.2%-62%) and 43.6% (95%CI 37.5%-49.7%) respectively, which were significantly lower than those in low Ki67 group which present as 92.9% (95%CI 89.0%-96.8%) and 88.3% (95%CI 82.4%-94.2%) respectively, and the difference was statistically significant (P<0.001).

**Conclusions:**

18% is the optimal cut-off value of Ki67 for predicting recurrence of NMIBUC. Ki67>18% is an independent risk factor for high recurrence rate of NMIBUC. This cut-off value can more accurately predict the risk of recurrence and has the potential clinical value for guiding the postoperative adjuvant treatment and follow-up strategy of NMIBUC.

## 1 Introduction

According to the latest statistics, bladder cancer (BCa) is one of the most common malignant tumors of the urinary system, with increasing mortality and morbidity (1). Of all types of BCa, non-muscular invasive bladder urothelial carcinoma (NMIBUC) is the most common pathological classification, accounting for 70% of BCa cases (2–4). Currently, transurethral resection of bladder tumor (TURBT) is the primary therapeutic approach for NMIBUC. Unfortunately, still approximately 60-70% of patients are reported to develop recurrence after surgery (5). Therefore, it is particularly critical to determine more indicators to accurately evaluate the postoperative prognosis of NMIBUC patients and guide management to reduce relapse.

With great advances in molecular biology and tumor biology, immunohistochemical markers have been considered for cancer prognostic assessments. Ki67, a nuclear antigen associated with the cell cycle, is significantly related to cell proliferation. It contributes to better estimation of the aggressive biological behavior of malignant tumor cells (6, 7). Many studies have found that the expression of the Ki67 protein is associated with tumor biological characteristics and prognosis (8, 9). In clinical practice, Ki67 is regarded as a clinical indicator to detect tumor cell proliferation activity and cancer prognosis because the expression of Ki67 can be easily detected by immunohistochemistry (7). For example, as reported in breast cancer, the high expression of Ki67 is helpful for identifying patients with poor prognosis (10). Similarly, Ki67, as a high-risk factor, is already used to assess the risk of endometrial cancer recurrence and guide the strategy effective adjuvant treatments (11). Additionally, some studies concluded that high-expression of Ki67 is associated with poorer prognostic indicators, including progression free survival (PFS), cancer specific survival (CSS), recurrence free survival (RFS), and overall survival (OS) in NMIBUC patients, which is particularly prominent in Europe and the United States; however, among Asian NMIBUC patients with recurrence and progression, the correlation between Ki67 expression and the above indicators is not obvious (12). In addition, many studies have shown that Ki67 may also be used as a significant predictor for postoperative recurrence of NMIBUC (13, 14). However, most clinicians only pay attention to the interpretation of positive or negative Ki67 expression (15). As a result, there is a lack of theoretical consensus on the criteria for defining positive or negative expression. Furthermore, there have been very few studies on the cut-off value of Ki67 to predict NMIBUC recurrence, which indicates that it is deficient to correctly assess the tumor consequence.

Therefore, this study aimed to evaluate the correlation between NMIBUC prognosis and Ki67 expression, as well as determine the optimal cut-off value of Ki67 to predict NMIBUC recrudescence, thus providing guidance for clinical treatments.

## 2 Materials and methods

### 2.1 Research population

The retrospective analysis has gathered medical information of the patients from January 2012 to January 2020 in the Yongchuan Hospital and the Second Affiliated Hospital of Chongqing Medical University. The pathological grading was evaluated by the WHO bladder cancer grading system (2004/2016): UCC-LG (short for low-grade urothelial carcinoma), UCC-HG (short for high-grade urothelial carcinoma), and PUNLMP (short for Papillary urothelial neoplasms of low malignant potential) (16). The inclusion criteria were as follows: (I) patients who primarily underwent the standard TURBT surgery; (II) the pathological type was NMIBUC (including the stage of Ta and T1) according to the Union for International Cancer Control (UICC); and (III) patients with sufficient medical information and requisite immunohistochemical markers, including CK20, P53, and Ki67. The cases that met the following criteria were excluded: (I) incomplete medical records; (II) patients failing to undergo TURBT surgery or receiving total or subtotal bladder resection; (II) the pathological type was nonurothelial tumor, including adenocarcinoma and squamous cell carcinoma; (III) bladder carcinoma *in situ* (stage Tis) (17): although this type belongs to NMIBC, it was also excluded due to its high risk of muscle invasion, poor differentiation, and high grade of aggression; (V) tumor metastasis; (VI;) no regular follow-up records: and (VII) Only a small number of patients in this study underwent BCG intravesical chemotherapy. These patients were excluded because they could not tolerate side effects such as cystitis, hematuria, etc. The procedures were conducted following the principles of the Declaration of Helsinki, and in accordance with the ethical standards of our hospital. All patients signed informed consent forms after the study protocol was fully explained.

### 2.2 Postoperative and follow-up

Approximately 60.4% (200/331) patients included in this study underwent bladder instillation chemotherapy. One of three drugs was selected: pirarubicin(40mg each time), epirubicin(50-80mg each time), or gemcitabine(1000mg each time). Due to some adverse effects of bacille Calmette-guerin vaccine such as hematuria, severe cystitis and etc., only a few patients were treated with BCG for bladder infusion chemotherapy, so they were excluded. Chemotherapy drugs are infused into the bladder through a urinary catheter and retained for 0.5-2 hours (according to the label). The participants were required to drain the bladder and drink less fluid before intravesical instillation. The appropriate solvent was selected according to the instructions of the drug. Standard TURBT surgery was performed in all participants. The standard strategy for follow-up was arranged from the operation date. The following subjects underwent foundational physical inspections, imaging and cystoscopy. The strategy adopted in this follow-up was: rechecking every three months within 1-2 years after surgery, rechecking every six months within 3-4 years after surgery, and rechecking every year after five years of surgery (18). The main imaging techniques for rechecking were urinary tract ultrasound and computed tomography (CT). During the follow-up, if the tests suggested the recurrence of BCa, further cystoscopy histopathological examination was needed (17); if clinical symptoms or imaging examination suggested no recurrence, cystoscopy was regularly conducted according to the established follow-up protocol. The deadline for follow-up was January 31, 2021.

### 2.3 Recurrence

During the follow-up, when clinical symptoms (such as hematuria) or imaging examination suggested BCa recurrence, secondary TURBT or cystoscopic biopsy was further performed to confirm tumor recurrence. The final result for recurrence should be identified by histopathological diagnosis (19). Recurrence is defined as any stage or grade of bladder urothelial carcinoma found in the bladder after surgery. Tumor progression is the occurrence of bladder wall myometrial invasion, regional lymph node invasion or distant metastasis during tumor recurrence. RFS was defined as the period from the initial surgical operation to recrudescence or the following deadline, and OS was defined as the time from the first operation to the last follow-up or death (20).

### 2.4 Immunohistochemistry analysis

All specimens were timely treated after an operation and were further processed following a standard and unified procedure in the pathology laboratory (21). Initially, specimens were fixed with formalin and further transformed into paraffin examples. Subsequently, according to eosin staining and hematoxylin, the lesion location was first identified. Next, CK20, P53 and Ki67 immunohistochemical analysis was conducted on an automatic immunostaining instrument (Leica Bond-Max, Milton Keynes, UK). Mouse monoclonal antibodies such as CK20 (clone Ks20.8), P53 (clone DO-7), and Ki-67 (clone 30-9) were used in immunohistochemistry. The tumor grade, lesion size, histological type, and infiltration depth were evaluated by our professional junior pathologists and then reviewed by our senior physician. The immunohistochemistry results were independently assessed by two expert pathologists. When the positive tumor cells were calculated the same, the evaluation was considered consistent; and when the initial assessment was different, the outcome was re-evaluated until reaching a consensus. The immunohistochemical parameters were as follows: CK20 cells without staining were negative (-); light yellow was weakly positive (1+); brownish yellow was moderately positive (2+), and brownish brown was strongly positive (3+) (18). The scoring system was as follows: < 5% P53 positive cells was scored as -; 5% ~ 25% was +; 26% ~ 49% was 2+; and ≥50% was 3+. Ki67 expression is shown by the percentage of positively stained cells (0% to 100%).

### 2.5 Statistical analysis

Data analysis was conducted using SPSS software (Version 26.0). Numeric variable was shown as the median and mean ± SD. The comparison was performed by t-test or rank-sum test. Categorical variables were shown as percentages and frequencies, and were compared by Chi-square test. The Youden index and receiver operating characteristic (ROC) curve were used to find the best cut-off Ki67 value. Multivariate and univariate Cox regression analyses were performed to verify the relationship between NMIBUC relapse and Ki67 expression. The survival analysis was shown in the Kaplan-Meier curve and Log-rank test. Using two-tailed tests, P<0.05 was considered statistically significant.

## 3 Results

### 3.1 Clinical and pathological features of patients and tumors

**Figure 1:**
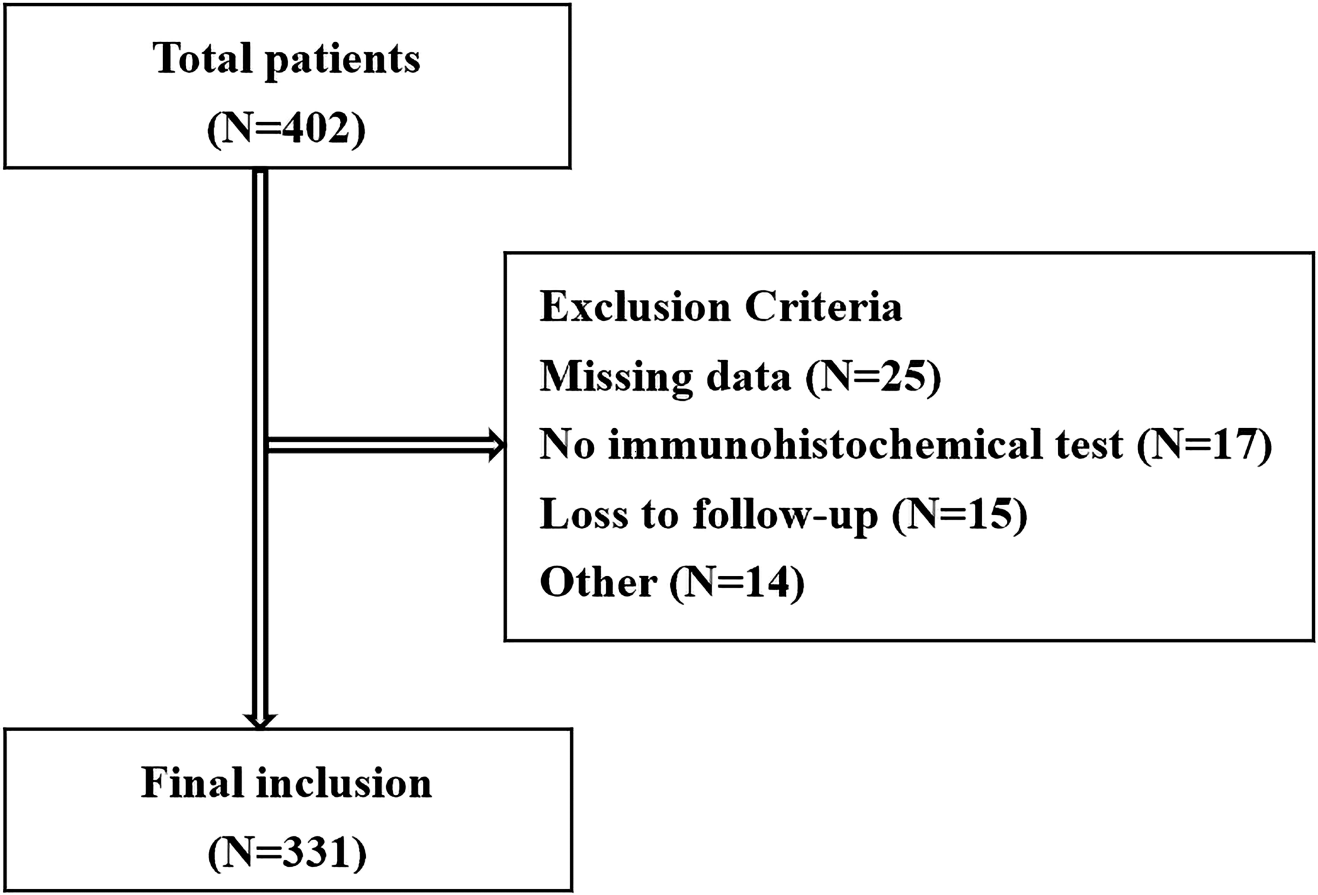
The flow chart for patient screening.

A total of 402 patients who were diagnosed with BCa receiving TURBT surgery were enrolled. According to the exclusion and inclusion criteria, 331 NMIBUC cases were included in the study **Figure 1**. Among these 331 patients, 138 (41.7%) reported recurrence, and 20 (6.1%) reported death. The Ki67 expression level ranged from 0% to 90% (median 30%). The median follow-up time and RFS were 41 months (4-102) and 27 months (4-89), respectively. Other clinicopathological data are shown in **Table 1**.

**Table 1:** Clinicopathological characteristics of patients.

Characteristics	N=331	%
Gender		
male female	256 75	77.3 22.7
Age (years)		
Median Mean Range	68 67.18 23-89	
Hypertension		
Yes No	113 218	34.1 65.9
Diabetes		
Yes No	40 291	12.1 87.9
Smoking history		
Yes No	193 138	58.3 41.7
Tumor grade		
PUNLMP UCC-LG UCC-HG	66 170 95	20.0 51.5 28.5
Tumor stage		
Ta	188	56.8
T1	143	43.2
Ki67		
median Mean Range	30 34.12 0-90	
P53		
- 1+ 2+ 3+	55 105 80 91	16.7 31.8 24.2 27.3
CK20		
- 1+ 2+ 3+	32 108 113 78	9.8 32.6 34.2 21.2
Tumor number		
1 ≥1	145 186	43.8 56.2
Tumor size (cm)		
<3 ≥3	186 145	56.2 43.8
Tumor location		
Vesicaltrigone Sidewall Anterior and posterior Others	192 26 22 91	45.1 7.8 6.5 40.6
IPPIT		
Yes No	200 131	60.6 39.4
Recurrence		
Yes No	138 193	41.7 58.3
Death Recurrence Other reason	20 8 12	6.1 2.4 3.6
DFS		
Median Mean Rank	27 31.57 4-89	
Follow-up time Median		
Mean Rank	41 45.56 4-102	

PUNLMP: Papillary urothelial neoplasms of low malignant potential; UCC-LG, Urothelial carcinoma-low grade; UCC-HG, Urothelial carcinoma-high grade; IPPIT, Immediate postoperative instillation therapy; DFS, Disease-free survival.

### 3.2 Optimal cut-off value of the Ki67 index

**Figure 2:**
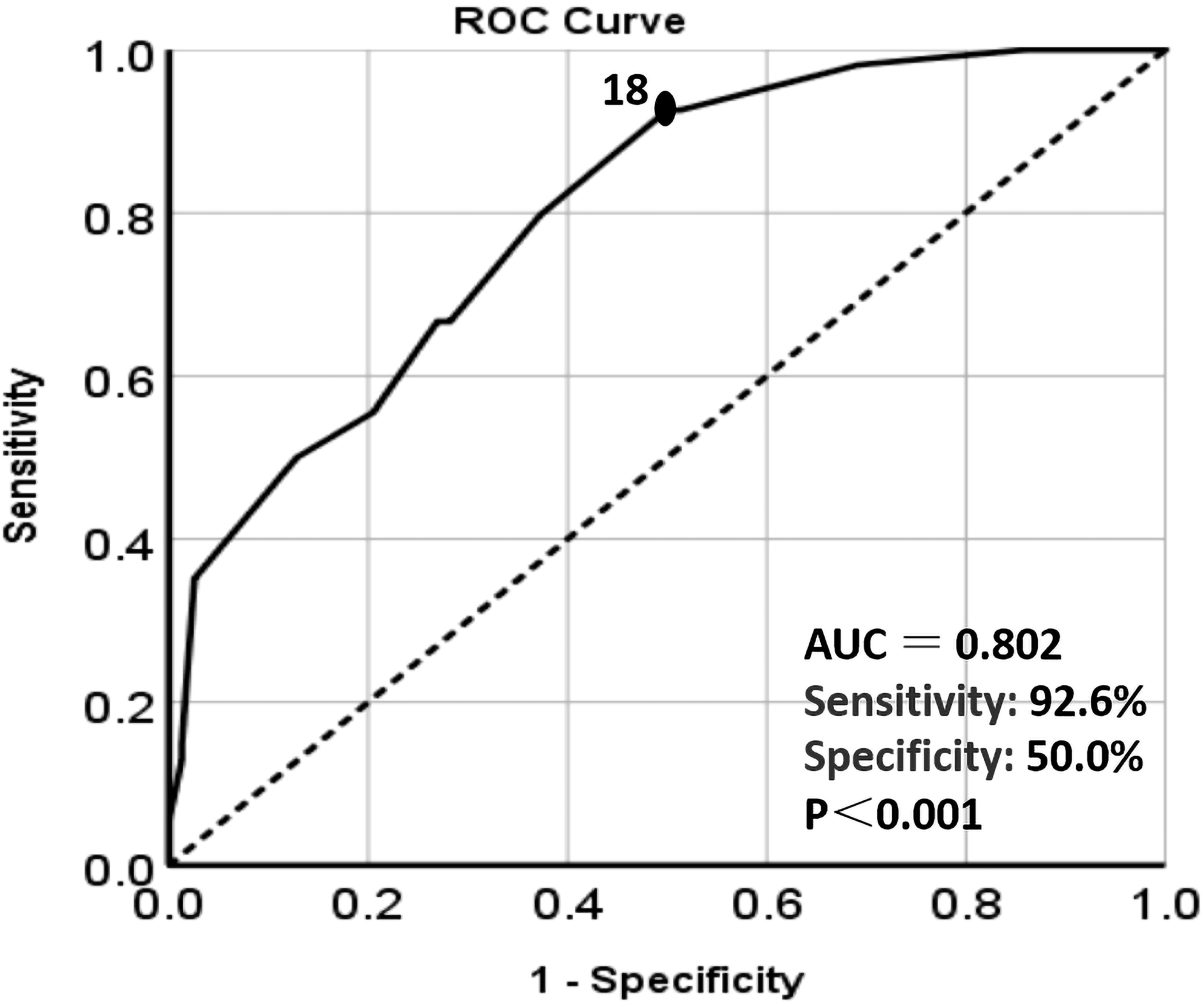
The ROC curve analysis of the relationship between Ki67 expression level and recurrence. The black dot: the area under the curve(AUC) at this point is the largest, which indicates the optimal cut-off value of Ki67. Dotted line: Reference line. Solid line: The ROC curve of Ki67.

The correlation between postoperative NMIBUC recurrence and Ki67 expression was calculated by the ROC curve. Finally, the optimal Ki67 cut-off value expression level to forecast relapse was recognized to be 18% [N = 331, area under the curve = 0.802, specificity 50.0%, sensitivity 92.6%, P <0.001, 95% confidence interval (CI)=0.728-0.875] **Figure 2**.

### 3.3 Prognostic factors of recurrence based on univariate and multivariate analyses

The clinical parameters and immunohistochemical markers that might cause recurrence were included in the univariate analysis. Gender (P=0.091), diabetes (P=0.331), age (P=0.143), smoking (P=0.128) and hypertension (P=0.545) did not correlate significantly with RFS. Thus, the parameters with P<0.05, tumor size and number, tumor stage, grade, immediate postoperative adjuvant therapy, CK20, P53, and Ki67, were further included in the multivariate analysis. Tumor grade (P=0.033), immediate postoperative adjuvant therapy (P=0.031), Ki67 (P=0.030), P53 (P=0.048) and CK20 (P=0.044) were shown to be significantly independent risk indicators for postoperative recurrence of NMIBC by multivariate analysis **Table 2**.

**Table 2:** The univariate and multivariate COX regression analysis of factors predicting non-muscle invasive bladder cancer recurrence.

Variables	Univariate analysis		P-value	Multivariate analysis		P-value
	Hazard ratio	95%CI		Hazard ratio	95%CI	
Tumor size	0.984	0.571-1.697	0.048	1.410	0.724-2.745	0.313
Tumor number	1.525	0.872-2.669	0.039	0.995	0.545-1.818	0.118
Tumor grade	6.132	3.219-11.680	0.001	2.284	1.071-4.874	0.033
Tumor stage	1.491	0.894-2.488	0.026	1.321	0.721-2.422	0.368
Ki67(≦18% vs >18%)	1.027	1.017-1.037	0.001	1.013	1.001-1.024	0.030
P53(0)	1.000		0.001	1.000		0.048
P53(1+)	1.189	0.358-3.952	0.778	1.116	0.309-4.030	0.867
P53(2+)	2.780	0.921-8.390	0.070	2.181	0.664-7.164	0.199
P53(3+)	5.373	1.876-15.387	0.002	3.425	1.056-11.110	0.040
CK20(0)	1.000		0.001	1.000		0.044
CK20(1+)	3.342	0.423-26.408	0.253	1.481	0.161-13.634	0.729
CK20(2+)	6.262	0.842-46.592	0.073	3.871	0.469-31.928	0.209
CK20(3+)	14.595	1.962-108.56	0.009	4.387	0.505-37.920	0.180
IPPIT	0.200	0.090-0.443	0.001	0.382	0.159-0.916	0.031

IPPIT, Immediate postoperative instillation therapy.

### 3.4 Comparison of clinicopathological features between groups

**Figure 3:**
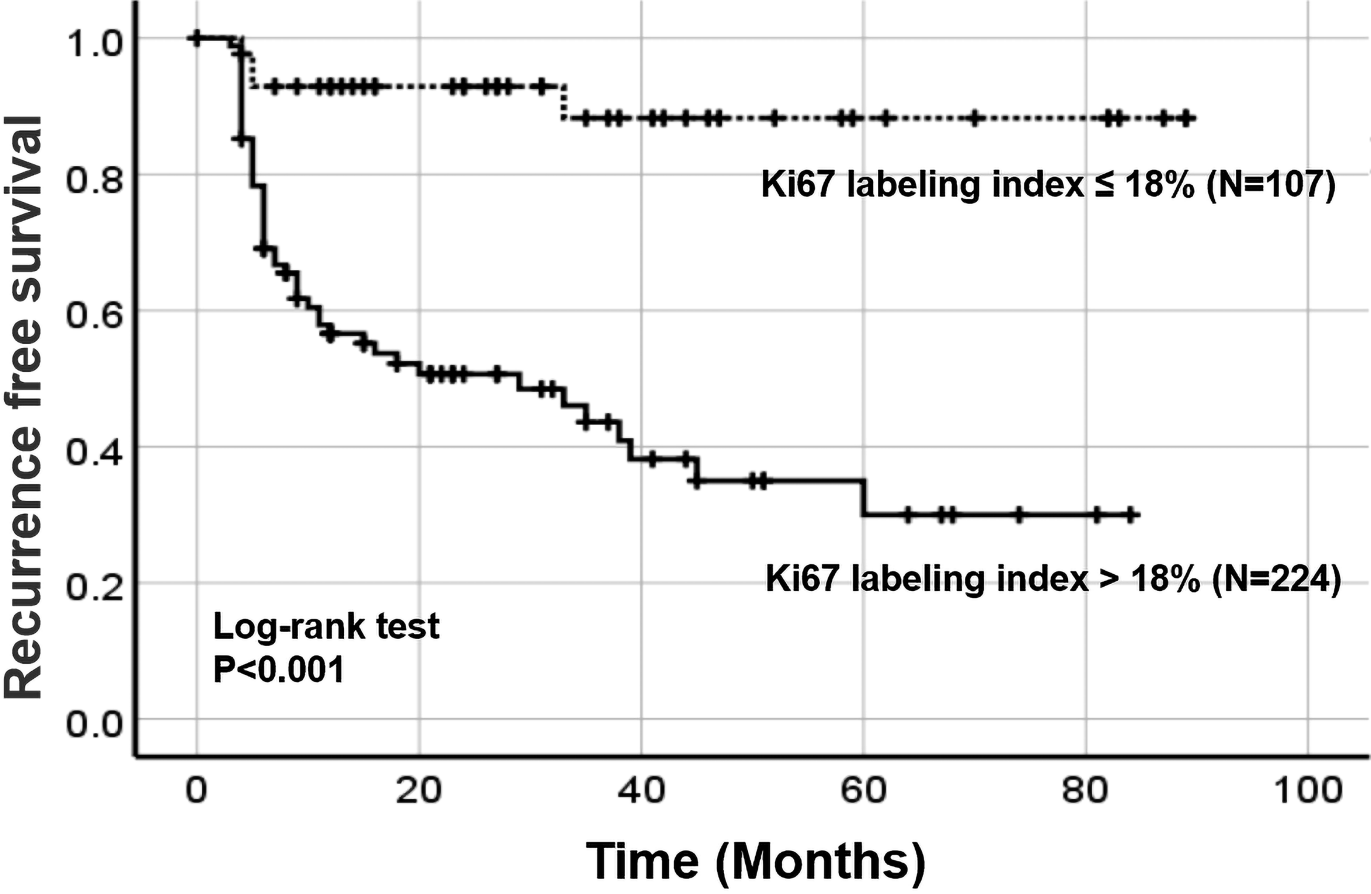
The recurrence-free survival of patients according to the optimal cut-off value of Ki67. Dotted line: The recurrence-free survival curve of the low Ki67 group. Solid line: The recurrence-free survival of curve the high Ki67 group.

According to the threshold of Ki67 (18%) concluded above, all patients were split into two groups: patients with Ki67>18% (high-Ki67 group) and patients with Ki67≦18% (low-Ki67 group). We found that the expression of high Ki67 was related to more aggressive characteristics: pathological grade (P=0.014), phase (P=0.001), the expression of P53 (P=0.019), immediate postoperative adjuvant therapy (P=0.001) and CK20 (P=0.001) **Table 3**. Furthermore, Kaplan-Meier analysis showed that the 3-year RFS was 43.6% (95% CI 37.5%-49.7%) in the high-Ki67 group and 88.3% (95% CI 82.4%-94.2%) in the low-Ki67 group, and the 5-year RFS were 30.0% (95% CI 22.7%-37.3%) and 88.3% (95% CI 82.4%-94.2%), respectively (P<0.001) **Figure 3**.

**Table 3:** Comparison of clinicopathological parameters of patients between low Ki67 group and high Ki67 group.

Variables	Ki67 labeling index ≤18% (n=107)	%	Ki67 labeling index >18% (n=224)	%	P-value
Gender					0.116
Male Female	75 32	70.1 29.9	181 43	80.8 19.2	
Age (years)					0.143
Mean ± SD	67.08 ± 10.596		65.14 ± 12.134		
Hypertension					0.894
Yes No	70 37	65.4 34.6	43 181	19.2 80.8	
Diabetes					0.490
Yes No	10 97	9.3 90.7	30 194	13.4 86.6	
Smoking history					0.124
Yes No	55 52	51.4 48.6	138 86	62.4 37.6	
Tumor grade					0.014
PUNLMP UCC-LG UCC-HG	20 51 36	18.7 47.7 33.6	46 119 59	20.5 53.1 26.4	
Tumor stage					0.001
Ta	75	70.1	113	50.4	
T1	32	29.9	111	49.6	
P53					0.019
- 1+ 2+ 3+	33 32 25 17	30.8 29.9 23.4 15.9	22 73 55 74	9.8 32.6 24.6 33.0	
CK20					0.001
- 1+ 2+ 3+	15 33 44 15	14.0 30.8 41.2 14.0	17 75 69 63	7.6 33.5 30.8 28.1	
Tumor number					0.124
1 ≥1	57 50	53.3 46.7	88 136	39.3 60.7	
Tumor size (cm)					0.431
<3 ≥3	55 52	51.4 48.6	131 93	58.5 41.5	
IPPIT					0.001
Yes No	65 42	60.7 39.3	135 89	60.3 39.7	

PUNLMP, Papillary urothelial neoplasms of low malignant potential; UCC-LG, Urothelial carcinoma-low grade; UCC-HG, Urothelial carcinoma-high grade; IPPIT, Immediate postoperative instillation therapy.

## 4 Discussion

**Figure 4:**
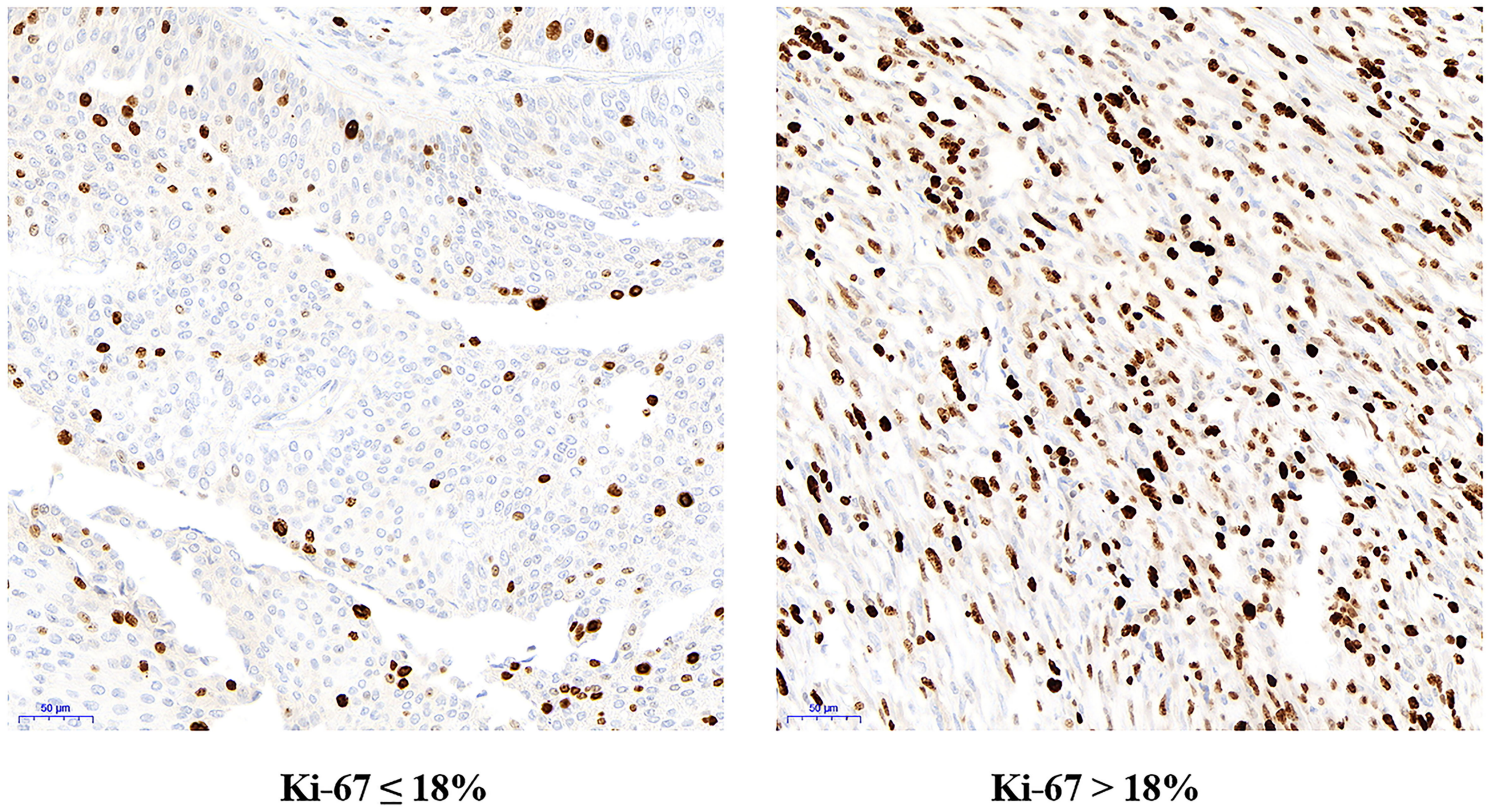
Immunohistochemical staining for Ki67 in low Ki67 group (Ki67 ≤ 18%) and high Ki67 group (Ki67 > 18%) under electron microscope.

As an indicator of cell proliferation, Ki67 is associated with the progression of cancer cells and has been universally used as a prognostic predictor to guide therapeutic decisions (22, 23). In this study, 18% was validated as the optimal cut-off value of Ki67 to predict the relapse of NMIBUC **Figure 4**. Even though the specificity was only 50% when the sensitivity was 92.6%, the maximum value of the Youden index assures the greatest specificity as well as the highest sensitivity, instead of only concentrating on the specificity or sensitivity. In addition, Ki67 expression was Strongly associated with tumor grade and stage as well as CK20 and P53 expression. This is because Ki67 expression indicates the proliferation activity of tumors. When the tumor is accompanied by more aggressive parameters such as advanced stage, worse grade, and higher expression of P53 and CK20, the expression of Ki67 is universally higher, which is consistent with other clinical studies (24). Ki67 overexpression plays a leading role in the prediction of bladder cancer recurrence, which indicates that the optimal cut-off value has the potential value for assessing the outcome of NMIBUC. Moreover, in the multivariate analysis, it was further concluded that Ki67>18% was an independent risk factor for prognosis. In other words, if the Ki67 value is higher, the recurrence risk is suggested to be higher. Moreover, we found that the RFS of patients with Ki67>18% was significantly lower than that of patients with Ki67 ≤ 18%, which suggests that more concern should be paid when Ki67 is over this cut-off value.

In this study, all included patients were treated with postoperative bladder infusion chemotherapy. According to the guidelines, for patients with low-risk BCa recurrence, only immediate infusion chemotherapy within 24 hours after surgery and early infusion therapy 4-8 times (once a week for 4-8 weeks) after surgery are needed; for patients with middle- or high-risk BCa recurrence, except for immediate postoperative bladder chemotherapy and early infusion chemotherapy 6-12 times (once a month, a total of 6-12 months), continuous infusion chemotherapy should also be completed (19). To define low-, middle- and high-risk recurrence patients, apart from traditional clinical parameters such as tumor number, size, tumor stage, grade and whether it is carcinoma *in situ*, the immunohistochemical marker Ki-67 can also be used as an effective tool (25, 26). For patients with Ki67>18%, it is suggested that active and comprehensive postoperative management be carried out, such as adding cycles of bladder infusion chemotherapy, extending the time of chemotherapy and performing frequent follow-up. In contrast, patients with Ki67≦18% incorporate favorable clinical features. Thus, to improve their quality of life, bladder infusion chemotherapy is not routinely recommended (27, 28).

The standard treatment regimen for NMIBUC is TURBT followed by adjuvant bladder infusion chemotherapy. Among the specimens obtained from TURBT, the acquisition of detrusor muscle (DM) tissue is extremely important (29). Studies have reported that the recurrence rates in patients with middle- and high-risk BCa recurrence range from 24% to 61% after one to five years of surgery (30). Currently, related studies are mainly about the impact of DM on prognosis in patients with middle- and high-risk BCa recurrence. For those with DM tissue deficiency, there is a greater risk of tumor residual, which significantly increases the possibility of tumor recurrence or progression (31). Comparatively, the recurrence rates in patients with low-risk recurrence (including Ta low grade) range from 15% to 31% after one to five years of surgery (30). Since tumor infiltration can be negligible in submucosal tissue, DM tissue plays a minimal role in postoperative recurrence in patients with low-risk recurrence (32).

Among other scoring systems for predicting postoperative recurrence of NMIBUC, the most commonly used is EORTC risk scores, in which the number of tumors, tumor size, prior recurrence rate, T category, carcinoma *in situ*, and grade are independent risk factors for the prediction of recurrence (32). Although this common model has high accuracy, it is limited to traditional clinical parameters and does not include immunohistochemical markers. In this study, Ki67 was an independent risk factor for predicting postoperative recurrence of NMIBUC. By introducing Ki67 into the above prediction model, its accuracy could be increased. Furthermore, this study also found that immediate postoperative adjuvant therapy, tumor stage, and the expression of P53 and CK20 are independent risk factors that affect postoperative recurrence, which is also consistent with other studies (33, 34). Nevertheless, this study illustrated that NMIBUC patients’ age, gender, smoking, tumor grade, tumor number, and size were not significantly related to postoperative recurrence. However, it does not deny the importance of these factors in predicting relapse; in fact, many studies have shown that they are important factors in predicting the prognosis of NMIBUC (35, 36). Therefore, when making the decision of adjuvant treatment, it is necessary not only to accurately evaluate the tumor’s biological behavior, but also to consider the level of immunohistochemistry such as Ki67 or according to the patients’ other clinicopathological indicators to reduce the risk of postoperative recrudescence and enhance prognosis (37).

Our study has some limitations. First, this study is a retrospective study, but it cannot be guaranteed that all data were completely and accurately recorded through the medical record system. Second, the postoperative specimens failed to undergo a unified review, but all specimens were analyzed in a unified standard procedure in the same authoritative institution. Additionally, the detection of Ki67 expression still lacks consensus. At present, as the method “hottest” is generally adopted, this study also used this hotspot scoring method to prove the predictive significance of the Ki67 index. However, there is still a necessity to establish a unified standard method for Ki67 expression. As a kind of nuclear antigen, the immunohistochemical marker Ki67 is related to cell proliferation, so it is closely associated with tumor progression. Regrettably, because of the small number of tumor-progression cases in this study, most patients with recurrence could only be diagnosed as recurrence at the time of diagnosis; only a small number of recurrent cases were judged as progressive and the value of Ki67 was positively correlated with tumor progression. Therefore, our team will strengthen the analysis of cases with tumor progression in subsequent studies.

## 5 Conclusions

In conclusion, the study demonstrated that the best cut-off value of Ki67 expression to predict NMIBUC postoperative recurrence is 18%. According to the cut-off value, clinicians can effectively develop treatment and follow-up strategies, which is of great clinical significance for promoting patient health.

## Data Availability

The data analyzed in this study is subject to the following licenses/restrictions: Patient privacy concerns. Requests to access these datasets should be directed to pijiangchuan@163.com.

## References

[1] Siegel RL Miller KD Jemal A. Cancer statistics, 2019. CA Cancer J Clin. (2019) 69:7–34. doi: 10.3322/caac.21551 30620402

[2] Tan WS Rodney S Lamb B Feneley M Kelly J. Management of non-muscle invasive bladder cancer: A comprehensive analysis of guidelines from the United States, Europe and Asia. Cancer Treat Rev. (2016) 47:22–31. doi: 10.1016/j.ctrv.2016.05.002 27231966

[3] Bray F Laversanne M Sung H Ferlay J Siegel RL Soerjomataram I. Global cancer statistics 2022: globocan estimates of incidence and mortality worldwide for 36 cancers in 185 countries. CA Cancer J Clin. (2024) 74:229–63. doi: 10.3322/caac.21834 38572751

[4] Zhang Y Li X Li X Zhao Y Zhou T Jiang X. Comprehensive analysis of cuproptosis-related long noncoding rna immune infiltration and prediction of prognosis in patients with bladder cancer. Front Genet. (2022) 13:990326. doi: 10.3389/fgene.2022.990326 36186475 PMC9515487

[5] Lu M Chen S Zhou Q Wang L Peng T Wang G. Predicting recurrence of nonmuscle-invasive bladder cancer (Ta-T1): A study based on 477 patients. Med (Baltimore). (2019) 98:e16426. doi: 10.1097/MD.0000000000016426 PMC6641864 31305463

[6] Miller I Min M Yang C Tian C Gookin S Carter D. Ki67 is a graded rather than a binary marker of proliferation versus quiescence. Cell Rep. (2018) 24:1105–12 e5. doi: 10.1016/j.celrep.2018.06.110 30067968 PMC6108547

[7] Inwald EC Klinkhammer-Schalke M Hofstadter F Zeman F Koller M Gerstenhauer M. Ki-67 is a prognostic parameter in breast cancer patients: results of a large population-based cohort of a cancer registry. Breast Cancer Res Treat. (2013) 139:539–52. doi: 10.1007/s10549-013-2560-8 PMC3669503 23674192

[8] Kloppel G La Rosa S. Ki67 labeling index: assessment and prognostic role in gastroenteropancreatic neuroendocrine neoplasms. Virchows Arch. (2018) 472:341–9. doi: 10.1007/s00428-017-2258-0 29134440

[9] Constantinou C Papadopoulos S Karyda E Alexopoulos A Agnanti N Batistatou A. Expression and clinical significance of claudin-7, pdl-1, pten, C-kit, C-met, C-myc, alk, ck5/6, ck17, P53, egfr, ki67, P63 in triple-negative breast cancer-a single centre prospective observational study. In Vivo. (2018) 32:303–11. doi: 10.21873/invivo.11238 PMC5905198 29475913

[10] Lashen AG Toss MS Ghannam SF Makhlouf S Green A Mongan NP. Expression, assessment and significance of ki67 expression in breast cancer: an update. J Clin Pathol. (2023) 76:357–64. doi: 10.1136/jcp-2022-208731 36813558

[11] Jiang P Jia M Hu J Huang Z Deng Y Lai L. Prognostic value of ki67 in patients with stage 1-2 endometrial cancer: validation of the cut-off value of ki67 as a predictive factor. Onco Targets Ther. (2020) 13:10841–50. doi: 10.2147/OTT.S274420 PMC7602913 33149602

[12] Tian Y Ma Z Chen Z Li M Wu Z Hong M. Clinicopathological and prognostic value of ki-67 expression in bladder cancer: A systematic review and meta-analysis. PloS One. (2016) 11:e0158891. doi: 10.1371/journal.pone.0158891 27410033 PMC4943634

[13] He Y Wang N Zhou X Wang J Ding Z Chen X. Prognostic value of ki67 in bcg-treated non-muscle invasive bladder cancer: A meta-analysis and systematic review. BMJ Open. (2018) 8:e019635. doi: 10.1136/bmjopen-2017-019635 PMC5905754 29666128

[14] Breyer J Wirtz RM Laible M Schlombs K Erben P Kriegmair MC. Esr1, erbb2, and ki67 mrna expression predicts stage and grade of non-muscle-invasive bladder carcinoma (Nmibc). Virchows Arch. (2016) 469:547–52. doi: 10.1007/s00428-016-2002-1 27514658

[15] Shi S Ma HY Sang YZ Ju YB Liu XY Zhang ZG. Expression and clinical significance of cmtm6 and pd-L1 in triple-negative breast cancer. BioMed Res Int. (2022) 2022:8118909. doi: 10.1155/2022/8118909 35845949 PMC9283057

[16] Humphrey PA Moch H Cubilla AL Ulbright TM Reuter VE. The 2016 who classification of tumours of the urinary system and male genital organs-part B: prostate and bladder tumours. Eur Urol. (2016) 70:106–19. doi: 10.1016/j.eururo.2016.02.028 26996659

[17] Cambier S Sylvester RJ Collette L Gontero P Brausi MA van Andel G. Eortc nomograms and risk groups for predicting recurrence, progression, and disease-specific and overall survival in non-muscle-invasive stage ta-T1 urothelial bladder cancer patients treated with 1-3 years of maintenance bacillus calmette-guérin. Eur Urol. (2016) 69:60–9. doi: 10.1016/j.eururo.2015.06.045 26210894

[18] Pi J Xiong Y Liu C Liao J Liu J Li C. A nomogram model to predict recurrence of non-muscle invasive bladder urothelial carcinoma after resection based on clinical parameters and immunohistochemical markers. J Invest Surg. (2022) 35:1186–94. doi: 10.1080/08941939.2021.2017080 34913802

[19] Babjuk M Burger M Compérat EM Gontero P Mostafid AH Palou J. European association of urology guidelines on non-muscle-invasive bladder cancer (Tat1 and carcinoma in situ) - 2019 update. Eur Urol. (2019) 76:639–57. doi: 10.1016/j.eururo.2019.08.016 31443960

[20] Jiang P Jia M Hu J Huang Z Deng Y Hu Z. A nomogram model involving immunohistochemical markers for predicting the recurrence of stage I-ii endometrial cancer. Front Oncol. (2020) 10:586081. doi: 10.3389/fonc.2020.586081 33585205 PMC7874072

[21] Yu X Guo S Song W Xiang T Yang C Tao K. Estrogen receptor alpha (Eralpha) status evaluation using rnascope in situ hybridization: A reliable and complementary method for ihc in breast cancer tissues. Hum Pathol. (2017) 61:121–9. doi: 10.1016/j.humpath.2016.12.005 27993577

[22] Fasching PA Gass P Haberle L Volz B Hein A Hack CC. Prognostic effect of ki-67 in common clinical subgroups of patients with her2-negative, hormone receptor-positive early breast cancer. Breast Cancer Res Treat. (2019) 175:617–25. doi: 10.1007/s10549-019-05198-9 30868391

[23] Liu J Ma C Li X Li A Wang Z. Circulating tumor cells correlating with ki-67 predicts the prognosis of bladder cancer patients. Int Urol Nephrol. (2023) 55:309–18. doi: 10.1007/s11255-022-03406-y PMC9859911 36334226

[24] Ziaran S Harsanyi S Bevizova K Varchulova Novakova Z Trebaticky B Bujdak P. Expression of E-cadherin, ki-67, and P53 in urinary bladder cancer in relation to progression, survival, and recurrence. Eur J Histochem. (2020) 64:3098. doi: 10.4081/ejh.2020.3098 32214283 PMC7118433

[25] Ravvaz K Walz ME Weissert JA Downs TM. Predicting nonmuscle invasive bladder cancer recurrence and progression in a United States population. J Urol. (2017) 198:824–31. doi: 10.1016/j.juro.2017.04.077 28433642

[26] Straccia P Fiorentino V Martini M Pierconti F. A systematic review and meta-analysis of ck20, cd44, ki67 and P53 as immunohistochemical markers in bladder carcinoma in situ. Actas Urol Esp (Engl Ed). (2022) 46:521–30. doi: 10.1016/j.acuroe.2022.08.013 36216762

[27] Sylvester RJ Oosterlinck W Holmang S Sydes MR Birtle A Gudjonsson S. Systematic review and individual patient data meta-analysis of randomized trials comparing a single immediate instillation of chemotherapy after transurethral resection with transurethral resection alone in patients with stage pta-pt1 urothelial carcinoma of the bladder: which patients benefit from the instillation? Eur Urol. (2016) 69:231–44. doi: 10.1016/j.eururo.2015.05.050 26091833

[28] Bosschieter J van Moorselaar RJA Vis AN van Ginkel T Lissenberg-Witte BI Beckers GMA. The effect of timing of an immediate instillation of mitomycin C after transurethral resection in 941 patients with non-muscle-invasive bladder cancer. BJU Int. (2018) 122:571–5. doi: 10.1111/bju.14124 29319922

[29] Dalbagni G. Editorial comment on: detrusor muscle in the first, apparently complete transurethral resection of bladder tumour specimen is a surrogate marker of resection quality, predicts risk of early recurrence, and is dependent on operator experience. Eur Urol. (2010) 57:849. doi: 10.1016/j.eururo.2009.05.048 19524355

[30] Mastroianni R Brassetti A Krajewski W Zdrojowy R Salhi YA Anceschi U. Assessing the impact of the absence of detrusor muscle in ta low-grade urothelial carcinoma of the bladder on recurrence-free survival. Eur Urol Focus. (2021) 7:1324–31. doi: 10.1016/j.euf.2020.08.007 32900676

[31] Dadiani M Bossel Ben-Moshe N Paluch-Shimon S Perry G Balint N Marin I. Tumor evolution inferred by patterns of microrna expression through the course of disease, therapy, and recurrence in breast cancer. Clin Cancer Res. (2016) 22:3651–62. doi: 10.1158/1078-0432.CCR-15-2313 26957561

[32] Sylvester RJ van der Meijden AP Oosterlinck W Witjes JA Bouffioux C Denis L. Predicting recurrence and progression in individual patients with stage ta T1 bladder cancer using eortc risk tables: A combined analysis of 2596 patients from seven eortc trials. Eur Urol. (2006) 49:466–5. doi: 10.1016/j.eururo.2005.12.031 16442208

[33] Bertz S Otto W Denzinger S Wieland WF Burger M Stohr R. Combination of ck20 and ki-67 immunostaining analysis predicts recurrence, progression, and cancer-specific survival in pt1 urothelial bladder cancer. Eur Urol. (2014) 65:218–26. doi: 10.1016/j.eururo.2012.05.033 22633802

[34] Poli G Cochetti G Boni A Egidi MG Brancorsini S Mearini E. Characterization of inflammasome-related genes in urine sediments of patients receiving intravesical bcg therapy. Urol Oncol. (2017) 35:674 e19– e24. doi: 10.1016/j.urolonc.2017.08.004 28888400

[35] Bosschieter J Nieuwenhuijzen JA van Ginkel T Vis AN Witte B Newling D. Value of an immediate intravesical instillation of mitomycin C in patients with non-muscle-invasive bladder cancer: A prospective multicentre randomised study in 2243 patients. Eur Urol. (2018) 73:226–32. doi: 10.1016/j.eururo.2017.06.038 28705539

[36] Messing EM Tangen CM Lerner SP Sahasrabudhe DM Koppie TM Wood DP Jr.. Effect of intravesical instillation of gemcitabine vs saline immediately following resection of suspected low-grade non-muscle-invasive bladder cancer on tumor recurrence: swog S0337 randomized clinical trial. JAMA. (2018) 319:1880–8. doi: 10.1001/jama.2018.4657 PMC6583489 29801011

[37] Dalkilic A Bayar G Kilinc MF. A comparison of eortc and cueto risk tables in terms of the prediction of recurrence and progression in all non-muscle-invasive bladder cancer patients. Urol J. (2019) 16:37–43. doi: 10.22037/uj.v0i0.4091 30120763

